# Enhancing research ethics and protections for uninsured and underinsured research participants in clinical trials in the USA

**DOI:** 10.1017/cts.2025.10192

**Published:** 2025-11-03

**Authors:** Assya Pascalev, Jane Otado, Priscilla Adler, Marc R. Blackman, Sarah Vittone

**Affiliations:** 1 Pellegrino Center for Clinical Bioethics, Georgetown University, Washington, DC, USA; 2 Departments of Philosophy and Interdisciplinary Studies, Howard University, Washington, DC, USA; 3 Department of Community Health and Family Medicine, College of Medicine, Howard University, Washington, DC, USA; 4 Institute for Clinical Research, Veteran Affairs Medical Center, Washington, DC, USA; 5 Departments of Medicine and Rehabilitation Medicine, Georgetown University, Washington, DC, USA

**Keywords:** Uninsured and underinsured study participants, clinical and translational research, vulnerable populations, justice, ancillary care

## Abstract

Inclusion of uninsured and underinsured (UUI) individuals in clinical research (CR) is necessary to ensure data quality, diversity, generalizability and fairness. Yet, in the USA, UUI persons tend to be excluded from CR. We conducted an ethical analysis of: the regulatory and ethics literature related to protections of, and duty to care for, research participants from vulnerable groups; the nature and scope of the ancillary health care obligations of researchers, and the applicable laws, regulations and practices concerning the care for UUI participants. We consider six examples illustrating the challenges of including UUI persons in CR. We note that addressing fully the challenges of UUI participation in CR requires comprehensive legal and health care reforms. We maintain that even in the absence of such reforms, researchers, study sponsors and Institutional Review Boards can and ought to adopt an inclusive approach to the recruitment of UUI individuals to improve data quality, diversity, generalizability and social justice. We propose such a systematic, proactive and ethically sound approach. It considers the medical and ancillary care needs of UUI participants, addresses them in the study protocol and budget, and includes referral to community health resources, follow-up support, and noting assistance in the research records.

## Introduction

Clinical research (CR) is central to the advancement of clinical and translational science. The intentional inclusion of uninsured and underinsured (UUI) research participants in CR in the USA is necessary to ensure data quality, generalizability and fairness. The notion “uninsured for health care” refers to individuals who lack any health insurance and primary care support. “Underinsured for health care” describes persons, whose health insurance is insufficient to cover their medical needs, e.g., it may include hospitalization but lack primary care. In the USA, such individuals have significant personal out-of-pocket spending for basic health needs and catastrophic illness, preventing many of them from seeking and receiving (adequate) health care. According to the Commonwealth Fund 2024 Biennial Health Insurance Survey, 9 percent of adults in the USA were uninsured, 12 percent had a gap in coverage during the prior year, and 23 percent were underinsured [1]. Accordingly, in the U.S. health care system, the opportunity to participate in CR is inherently related to a person’s ability to secure access to health care. This puts UUI individuals at a significant disadvantage regarding their ability to obtain equal access to CR. Consequently, research participants with insufficient health insurance may experience discriminatory exclusion from clinical studies. The under-representation of such individuals in CR impairs innovation, hinders generalizability of evidence, and undermines trust [2–4]. Therefore, promoting the inclusion of UUI participants from varying socio-economic, geographic and educational backgrounds is essential to inform CR from study design to dissemination of results so as to generate valid, generalizable evidence that meets the needs of an increasingly diverse population and addresses health inequities in the USA [5]. Yet, due to the lack of (sufficient) health insurance to cover their medical needs during the study, including UUI individuals in clinical trials puts them at increased risk of harm. This in turn poses major ethical and logistical challenges to their participation, and undermines fairness and protections as the costs of medical care during a study usually are not covered by the study sponsor but are paid for by the participant’s own health insurance.

The UUI status of study participants also poses major ethical challenges for CR related to health equity and protections [6]. When research participants lack sufficient health insurance, they become more vulnerable to health risks and may experience discrimination and exclusion from clinical studies. As a result, the lack of adequate health insurance becomes a barrier to research participation for members of certain socio-economic groups and results in inconsistencies in meeting their medical needs. Moreover, the exclusion of these groups from CR severely limits diversity, which compromises the generalizability of the findings and the quality of translational science. Therefore, including UUI individuals in CR is scientifically, socially and ethically important. While it is important to acknowledge that participants from UUI groups are susceptible to increased risks, vulnerabilities and exploitation due to their socio-economic status e.g., low levels of health literacy and limited or no access to health care, their inclusion in research is essential for understanding and improving the health and overall well-being of these groups and as a matter of social justice and fair distribution of benefits and burdens in CR [7].

The purpose of this paper is to highlight the current issues surrounding UUI populations in the U.S.A so as to increase their participation in CR, and to offer specific guidance and recommendations to all parties involved in CR. We consider six examples illustrating the challenges of including UUI persons in CR. We demonstrate that researchers and Institutional Review Boards (IRBs) have an obligation to advocate for, and ensure, the inclusion of diverse populations when recruiting participants for CR, as diversity is essential for the rigor and generalizability of the science, and is required to fulfill the ethical principles of respect, reciprocity and solidarity. Researchers and study sponsors have a moral obligation to plan for and address the medical needs of all participants in CR, including UUI persons, as a matter of social justice and data quality. Clinical researchers should include a plan for medical and ancillary care of UUI participants [8]. Ancillary care is defined as “care that research participants need that is not essential to make the research safe or scientifically valid, and is not needed to remedy injuries that eventuate as a result of the research project itself” [9]. At a minimum, researchers and study sponsors should provide UUI participants with information regarding existing community health resources and follow-up support [10,11]. To fulfill the ethical requirement for safe and equitable treatment of all participants in CR in the USA, clinical researchers should strive to secure dedicated funds in the study budget to assist those UUI participants who may need medical care.

## Methods

We conducted an ethical analysis of the challenges related to UUI participants’ lack of sufficient health insurance. Ethical analysis is a method which considers different viewpoints, identifies relevant ethical principles and applies them to the issues at hand, weighing responsibilities, actual and potential consequences including harms and benefits, and considers the impact on interested parties to inform guidance. In our ethical analysis we reviewed (1) the regulatory and ethics literature concerning protections and duty to care for research participants and potential participants who are UUI members of vulnerable groups and (2) considerations related to the nature and scope of ancillary care obligations of researchers.

## Results

Health care and CR are inextricably inter-related. They share the same mission: to improve health. Therefore, obligations to the principles of beneficence, respect for persons, and justice apply similarly. Due to the innate risks of CR for research participants, researchers ought to employ a heightened sense of protection of UUI individuals while ensuring their inclusion, as UUI research participants have unique vulnerabilities and their inclusion is essential to ensure the scientific quality of CR. Yet while the need to include UUI participants is recognized, the obligations to fully engage them and provide resources for their participation in CR have not been articulated in policy and as a matter of practice.

The Belmont Report [7] sets forth specific guidance on the inclusion of vulnerable research participants and identifies inclusion risks for such participants due to their dependent status. Although these risks remain and the concerns related to their inclusion are justified, we note that the exclusion of UUI individuals in the USA due to lack of health resources may contribute to social injustice.

The Common Rule [12] and FDA [2] regulations require IRBs to give special consideration to protecting the welfare of vulnerable participants but do not provide specific guidance regarding the responsibilities of researchers to address the medical needs of UUI research participants. Institutions such as the NIH, FDA and ORI require researchers to recruit participants from diverse socio-economic backgrounds in order to strengthen the scientific results. Yet, the recruitment of diverse participants is still a challenge for CR in the USA despite current efforts [13–15].

The aforementioned responsibility, the evolving social landscape, and ethical considerations call for rethinking, reevaluating and instituting the necessary logistics allowing for inclusion and protections of UUI populations [2,16]. This necessitates a shift in how we apply the principles of the Belmont Report, with an increased emphasis on the principle of justice. Researchers need increased understanding and guidance regarding the scope of their ethical obligations to UUI participants and how to translate this knowledge into action through recruitment of this population. We maintain that researchers have an ethical duty to address the needs of UUI participants as a matter of justice, respect for persons, and beneficence, as required by the Belmont Report [7]. Moreover, research ethics has moved beyond the three principles of Belmont to include the principles of solidarity and reciprocity, which offer additional value to the research enterprise [17,18]. Solidarity as an ethical principle encourages participants to accept risk in research, yet adds a reciprocal investigator responsibility to protect and advocate for research participants. This necessary relationship between investigator and study participant has evolved into a more collaborative approach to research, supplanting the view that research participants serve merely as a means to an end. Investigators are expected to conduct their research with an increased awareness of the participant’s full life experience, not only that related to the study in question. This awareness presupposes a more robust responsibility, a reciprocity to protect, advocate, and consider the impact of the research on the participant’s lived experience.

The traditional model of the relationship between the researcher and research subject, which is primarily focused on the autonomy of the latter, does not adequately represent the current understanding and necessary relationships of researchers to their study participants. These relationships ought to be informed by an awareness of the historic injustices and the resulting socio-economic disenfranchisement of substantial segments of American society, which are root causes of inequality in American society, including health disparities, one aspect of which is the lack of adequate health insurance. Correcting the injustices requires a broader societal effort. In this context, the principle of justice becomes central to conducting ethical CR. Research centers, IRBs and individual researchers have an obligation to contribute to the corrective action through inclusion efforts. Excluding certain groups from study recruitment based on their potential medical needs is antithetical to the ethical and scientific obligations in CR [19]. In evaluating recruitment efforts, IRBs should make a deliberate effort to confirm recruitment strategies for UUI participants [16]. They should be mindful of the discriminatory nature of identifying health insurance or lack thereof as an exclusion criterion, and should ask investigators for further explanations, as needed. This is particularly important for CR programs located in communities in the USA with a high prevalence of low-income patients and immigrants, many of whom may be UUI individuals.

While these ethical and professional obligations have been identified in the literature and through regulatory protections, there are additional barriers for CR stemming from the legal restrictions, and the current structure and funding of healthcare in the USA. Under current US law, study sponsors are legally prohibited from providing participants with assistance, which is not directly related to the research study. Sponsors who may be willing to offer such assistance may run afoul of the Anti-Kickbacks (AKB) Statute (42 U.S.C. § 1320a-7b(b)) prohibiting pharmaceutical companies and other commercial sponsors from providing payments or benefits to healthcare providers that go beyond legitimate research-related expenses. Sponsors are allowed to cover only expenses deemed “legitimately necessary” for conducting the clinical study. Furthermore, under Medicare, it is illegal to cover patients’ co-pays [20]. Thus, having a research sponsor reimburse co-pays could cause legal issues for researchers and underinsured participants who are on Medicare. These legal restrictions along with their ethical underpinnings are intended to prevent ethical misconduct by providers and sponsors, yet they have not evolved with the changing social landscape.

## Discussion

It is important to ensure that the medical needs of study participants from UUI and other underrepresented groups are properly addressed, that all necessary safeguards and support are in place, and that they are appropriate to the nature of the study and the potential risks to the participants.

Below, we present six examples to illustrate challenges to this mandate. Due to space limitation, we summarize the examples in Table One and discuss in greater detail the first three cases. A discussion of the last three examples is included in the Appendix.

The first example concerns types of CR, e.g., cancer studies, which require that all participants have access to standard of care as part of the research protocol [21]. Consequently, individuals who are uninsured yet otherwise eligible to participate in a cancer study would not be able to receive the standard treatment and, thus, would not be able to participate in a cancer study. This adds to the disparity of the recruited population and undermines justice by limiting fair access to research for extraneous, socio-economic reasons rather than science-based criteria [22].

Second, post-trial access to the resulting clinical treatments is a well-recognized benefit of research participation. Yet, this creates an ongoing ethical concern for UUI participants, who would not have this access because of their UUI status [23,24]. The current systems of CR and healthcare in the USA include high individual cost (out of pocket), which severely limits access to resulting costly pharmaceuticals for those participants who accepted the risk of trials contributing to development of new effective treatment(s) that may otherwise benefit them. We maintain that all participants including UUI must be allowed to share in the benefit directly. Ethicists acknowledge this direct benefit obligation [23] and have demonstrated it in regard to engagement in global research, especially in under-resourced countries [25]. Surely, we have a similar obligation to our domestic study participants. Yet this has not been fully recognized and it is unclear how benefit sharing can be achieved as a matter of practice in the USA. Therefore, this potential limitation to post-trial access by UUI participants must be disclosed early in the informed consent process.

Third, UUI persons with inherent vulnerability may be unwilling to disclose their health insurance status during study enrollment out of fear that this may exclude them from participation. UUI persons may experience a power imbalance in their relationship with the research team, limited also by their insufficient understanding which impacts their ability to advocate for themselves effectively. Therefore, how research teams manage initial recruitment, explaining risks, including necessary access to healthcare resources during the CR is important to enable this participant advocacy. Research teams, in an effort to protect UUI participants during eligibility screening, may err too heavily on the obligation to protect them and may risk becoming paternalistic instead of promoting UUI individuals’ interest in participating in the research.

As the examples illustrate, the value of creating an open safe environment for all participants to disclose their UUI status cannot be understated. Community Resources available must be known and readily shared to maintain ethical obligations to participants and their safety and ongoing enrollment. Connecting participants to resources will address even our post-trial direct benefit obligation [23] which has been demonstrated with regard to engagement in global research in under-resourced countries [25]. Finally, increased awareness and new approaches need to be explored to ensure advocacy, inclusion and protection for UUI individuals in CR. Research teams should strive to be aware and provide access to community services for all participants’ medical care needs. Direct support and education for those who disclose their UUI status is required.

### Recommendations

The PIs and IRBs should anticipate not only potential medical care needs related to the study protocols but also participants’ possible ancillary care needs. PIs should identify and document these potential needs throughout the study and suggest that funding be aggregated for a larger research participant pool. Dedicated funding to cover ancillary care responsibilities should be negotiated in the initial and ongoing research budgets, including available community health resources. Institutions and sponsors should be encouraged to consider ancillary care duties an essential component of conducting ethical research. Yet, as noted above, under current US law, sponsors have a legal restriction from providing assistance for participants that is not directly related to the research study and Medicare prohibits covering patients’ co-pays [20]. Until these legal restrictions are removed, at a minimum, research centers should develop a list of existing community-based resources and should make it available to UUI participants [17,26].The list should be established, maintained and continually updated by institutional regulatory and ethics specialists, in collaboration with community health leaders. It should be made available to researchers and study teams to assist UUI participants to access care for medical needs falling outside the study. When a UUI participant is unenrolled for medical reasons or due to study noncompliance, follow-up support should be provided in the form of an interview with the study coordinator to ensure that the participant understands the objective basis of the decision to unenroll and to identify any urgent needs the participant might have. In case of such needs, the unenrolled participant should be referred to a designated member of the research team for information on local resources to meet those needs and to ensure that the participant is supported and does not feel abandoned. The assistance given to unenrolled participants should be recorded and made part of their research record.

Special attention should be paid to the informed consent process due to the particular risks associated with UUI individuals during screening, enrollment and study participation. It is vital to ensure that UUI participants are fully informed of: (1) the risks, benefits, qualifying and disqualifying criteria, (2) the need to comply strictly with study protocols, (3) the goals of CR, and that the research is intended to advance knowledge about conditions that affect their population but is not designed to benefit them individually, (4) what medical care might be required in case of side effects and adverse events, and whether or not it is covered by the study, and (5) the resources and assistance available at no cost to the participants to meet medical needs not covered by the study. The scope and nature of the assistance should be specified. Special care should be taken to prevent any possible therapeutic misconception and to ensure that UUI participants are fully aware that their study participation is intended to produce generalizable scientific knowledge and not to deliver therapeutic benefits to the individual participant. Research teams must be aware of their potential bias to protect UUI persons by declining to enroll them instead of advocating for their inclusion in CR especially where adequate community services are available and can be identified. Inclusion and exclusion criteria should be explained clearly and participants should be informed that these are science-based decisions, including the decision to withdraw a participant from a study. It should be made clear that such decisions are rendered purely on scientific grounds and are not related to the participant’s personal, racial, ethnic, legal or socio-economic status. Participants should be informed of the resources and assistance they can receive in case of a medical need, and the scope and nature of the assistance should be specified.

UUI persons have a greater risk of experiencing unaddressed ancillary health issues. Under some circumstances, such as an acute illness, research participant may be prevented from further participation in the research. and, there would be a need for unmasking for safety reasons. Wherever feasible, the PI should discuss the specific situation with a research participant advocate and/or an IRB member and communicate the decision to the participant in a clear, understandable and culturally sensitive manner. While the informed consent document includes details related to study withdrawal, PIs should consider reminding participants at the point of unmasking that the latter leads to study withdrawal. Participants or their legally appointed representatives should be given the opportunity to ask questions and receive information to their satisfaction in a respectful and culturally sensitive manner so as not to leave a sense of abandonment.

IRBs are responsible to ensure the safe, ethical and equitable treatment of all participants in CR. Therefore, it is their obligation to ensure that UUI individuals are not excluded from CR, and that the study team and sponsors (institutional, federal, industrial, philanthropic, etc.) plan for the provision of possible research-related medical needs of UUI study participants. IRBs should ascertain that there are adequate resources to ensure the care and safety of participants at all phases of the research project; and anticipate the ancillary care responsibilities that may arise. They should be considered a necessary part of ethical research which supports diversity under the principle of justice. We suggest that, as part of a study’s feasibility assessment, the IRBs include UUI as a category similar to gender, ethnicity and race. It should require investigators and sponsors to plan for ongoing and future UUI supports and include these in the initial and continuing review. While currently this may be seen as falling outside the purview of IRBs, the obligations to protecting and advocate must require this to meet the overall mission of IRBs.

PIs should have an enlightened relationship with research participants and consider the needs of each participant as a whole person. This entails (1) anticipating not only the medical care needs of UUI research participants which are directly necessary for the study but also their ancillary care needs; (2) developing relationships with regulatory and ethics specialists and community providers; (3) ensuring that funding for related medical care and ancillary care are available; (4) ensuring that all staff members are properly trained in the procedures for obtaining informed consent and addressing the medical needs of UUI participants; (5) ensuring that participants understand that the research is designed to advance knowledge and may not benefit them individually, (6) providing for communication and dissemination of general and individual research findings to research participants [27], and (7) wherever feasible, the PI should discuss the specific situation with the research participant advocate and/or an IRB member and communicate the decision to the participant in a clear, understandable and culturally sensitive manner. While the informed consent document includes details related to study withdrawal, PIs should consider reminding participants at the point of unmasking that it leads to study withdrawal. Participants or their legally appointed representatives should be given the opportunity to ask questions and receive information to their satisfaction in a respectful and culturally sensitive manner so as not to leave a sense of abandonment.

Study Coordinators should consult with the PI and: (1) answer questions the participant may have related to unmasking and/or withdrawal from the study; (2) address, in consultation with the PI, concerns of the participant related to the study, medical needs or withdrawal; (3) provide the participant with a list of local medical care resources available for this population.

We recommend that research institutions create an independent Regulatory, Ethics Knowledge Support (REKS) Group which is a collaborative team of experts working together to address the myriad concerns related to research participants and to recommend education and policy changes to support CR. The REKS groups can be constituted as subgroups of IRBs, institutional compliance offices and/or other internal or external research oversight bodies. It should include Research Participant Advocates (RPAs), who are independent from the research team, but part of the REKS. They can be a valuable resource to protect the interests of UUI participants [28]. The RPA acts as an unbiased observer, and serves as a resource with a voice for the study participants’ rights, protections, research safety and responsible conduct of research.

As noted, members of a Community Engagement Group should collaborate and work with the research community and REKS to create and maintain an up-to-date list of community-based resources to address potential medical needs of UUI participants. Community Engagement staff should consult with the PI and the REKS team about concerns raised by UUI participants or those who have been withdrawn. The Community Engagement team should work closely with a Community Advisory Board and seek its input on the most appropriate ways of addressing concerns of inclusion and protection for UUI participants.

Sponsors need to provide appropriate mechanisms for funding UUI so that they can be included in CR and contribute to the science. Several pharmaceutical sponsors have recently added line items to budgets to cover medical needs of UUI participants should they be recruited to clinical studies. This budget line item, however, is not usually included in non-pharmaceutical studies or federally funded CR. For example, investigator-initiated studies, foundation grants, and non-funded research studies do not have the funds to cover these medical costs. As discussed above, under current US law, sponsors are legally restricted from covering expenses that are not seen as legitimately necessary for conducting the clinical study, and underinsured Medicare recipients are barred from receiving assistance with their co-payments.

## Conclusions

Lack of adequate health insurance in the USA limits UUI individuals’ access to clinical trials and reduces diversity in CR by creating potential financial barriers to research participation for members of socio-economically and medically underserved groups. This limitation poses a risk of discriminatory exclusion and leads to inter-institutional inconsistencies in meeting the medical needs of this vulnerable population during a clinical study. To address these challenges, we propose an inclusive, systematic, proactive and ethically sound approach designed to plan for the medical needs of UUI participants and to identify existing community health resources which could address their medical needs and encourage members of UUI groups to participate in CR. We propose that researchers, IRBs and study sponsors adopt such an inclusive approach towards the known and potential medical needs of UUI participants, and plan for their ancillary care should the need for such a care arise during a study. Some of our specific recommendations may appear aspirational and likely to face implementation challenges, but these difficulties do not relieve us from the evolving ethical responsibilities to include UUI individuals in CR. Including diverse participants in CR ensures data quality, fair distribution of benefits and burdens, and social justice. This is why further discussions and concrete steps to address the inclusion, rights, needs and protections of UUI participants in CR in the USA should become part of the planning and implementation of all CR and should be incorporated in the national research agenda. Addressing fully the issue of UUI participation in CR in the USA requires radical steps: from a major restructuring of the way health care is being provided and covered to a legal reform of the AKB statute and the relevant Medicare legal restrictions. Yet, as our analysis indicates, even in the absence of such reforms, there are still concrete steps that could be taken by researchers, IRBs and sponsors to promote and increase the participation of UUI individuals in CR.

**Table 1.** Challenges of enrolling uninsured and underinsured (UUI) individuals in clinical research in the USA

Type of UUI issue	Unique impact
Access to insurance and standard of care (SOC) as part of the study protocol [21]	UUI would not be eligible if not receiving SOC, therefore adds to disparity in recruitment, undermines justice, limits fair access [22]
Post-Trial access to treatment is an obligatory benefit to participants.	UUI would not have post-trial access without insurance. [23,24] High out of pocket costs would be unrealistic posing a barrier.
Self-Disclosure of UUI status	UUI persons with inherent vulnerability may be unwilling to disclose status for fear of exclusion. Research team may not enroll eligible participants in order to minimize risks and in a heightened sense of paternalism.
Loss of insurance during study participation	A participant may lose health insurance during the course of the research and may fear disclosure of UUI status will lead to withdrawal and loss of study care.
Therapeutic misconception	This is uniquely heightened for UUI participants due to their invaluable access to beneficial clinical care through the research visits. Intentional and ongoing education is necessary.
Risk of experiencing ancillary health issues	UUI persons lacking primary care may have poorer overall health status leading to increased risk of ancillary health issues than insured participants. UUI may be prevented from enrolling due to poor health failing our obligation to social justice and risking social abandonment.

## Supporting information

10.1017/cts.2025.10192.sm001 Pascalev et al. supplementary material Pascalev et al. supplementary material

## Supplementary material

The supplementary material for this article can be found at https://doi.org/10.1017/cts.2025.10192.
