# Supplementary material for: Enhancing research ethics and protections for uninsured and underinsured research participants in clinical trials in the USA
Source: J Clin Transl Sci. 2025 Nov 3;9(1):e285. doi: 10.1017/cts.2025.10192 (PMC12780799; doi:10.1017/cts.2025.10192)
Supplement: Pascalev et al. supplementary material [file S2059866125101921sup001.docx]

**Appendix**

**Enhancing Research Ethics and Protections for Uninsured and Underinsured Research**  **Participants in Clinical Trials** **in the USA**

Assya Pascalev, PhD^1a,2a^, Jane Otado, PhD^2b^, Priscilla N. Adler, MBA^3a^, Marc R. Blackman, MD^1b^, Sarah Vittone, DBe^1a^

**Some challenges of enrolling UUI individuals in clinical research in the USA: Discussion**

When a participant **loses health insurance during the course** of the research, especially in studies that require standard of care, s/he might be exposed to increased risk and burdens and increased out-of-pocket expenses. Without additional community-based health resources, this participant with a new UUI status, may be summarily withdrawn from the study by the research team. Further, the participant may not disclose her/his changed UUI status due to fear of being withdrawn and losing access to the care currently provided in the course of the study.

Furthermore, the general risk of **therapeutic misconception**, the belief that research participation would have individual benefit, which is heightened for UUI participants. These participants have a unique experience being UUI and may intend with their participation to gain access to health services for the purpose of individualized benefits. This idea that CR itself provides access to routine health care must be clearly dispelled.

Additionally, UUI persons are more likely to have less access to primary care and may have poorer overall health status and greater risk of experiencing ancillary health issues. Because of this, under some circumstances, such as an acute illness or related comorbidities, UUI individuals may be prevented from enrollment, or from continuing participation when already enrolled, at a higher rate than insured participants. As an additional failure in our obligation to social justice, the UUI may experience abandonment by the research enterprise in the USA, which compounds their UUI experience with the lack of health care services in the USA.

The above examples demonstrate that increased awareness and new approaches need to be explored to ensure advocacy, inclusion and protection for UUI individuals participating in CR.
